# Adult attention-deficit/hyperactivity disorder: Associations between subtype and lifetime substance use – a clinical study

**DOI:** 10.12688/f1000research.6780.2

**Published:** 2016-10-19

**Authors:** Michael Liebrenz, Alex Gamma, Iliyan Ivanov, Anna Buadze, Dominique Eich

**Affiliations:** 1 Institute of Forensic Medicine, Department of Forensic Psychiatry, University of Bern, Bern, 3012, Switzerland; 2 Department of Psychiatry, Mount Sinai School of Medicine, New York, NY, 10029, USA; 3 Psychiatric University Hospital, Research Group on ADHD, Zurich, 8032, Switzerland

**Keywords:** Attention deficit hyperactivity disorder (ADHD), subtype, presentation, substance-use disorder, cocaine, dual-diagnosis

## Abstract

ADHD is the one of the most prevalent childhood disorders and has been associated with impairments persisting into adulthood. Specifically, childhood ADHD is an independent clinical risk factor for the development of later substance use disorders (SUD). Moreover, adults who meet diagnostic criteria for ADHD have shown high rates of comorbid SUDs. Few studies, however, have reported on the relationship between ADHD subtypes and SUD in adult samples. The purpose of this study was to characterize a clinical sample of adults with ADHD and to identify possible associations between ADHD subtypes, lifetime substance use, and if ADHD subtypes may be preferentially associated with specific substances of abuse. We recruited 413 adult ADHD patients, performed an evaluation of their ADHD and conducted an interview on their use of psychotropic substances. Complete data was obtained for 349 patients. Lifetime substance abuse or dependence was 26% and occasional use was 57% in this sample. The inattentive subtype was significantly less likely to abuse or be dependent on cocaine than the combined subtype. Our findings underscore the high rate of comorbidity between substance use and ADHD in adults. The more frequent abuse/dependence of cocaine by adult patients with hyperactive-impulsive symptoms should be kept in mind when treating this patient group.

## Introduction

Attention-deficit/hyperactivity disorder (ADHD) is a complex neuropsychiatric syndrome that is common not only in childhood and adolescence, but in adulthood ^1– 4^. It is characterized by symptoms of inattention (distractibility), hyperactivity, and impulsivity, which all contribute to significant psychosocial impairment in affected individuals of all age groups ^5– 7^. In order to make a diagnosis of ADHD, the two diagnostic manuals, the American Psychiatric Association’s (APA) Diagnostic and Statistical Manual of Mental Disorders Third Edition Revision (DSM-III-R) and the World Health Organisation’s (WHO) International Statistical Classification of Diseases and Related Health Conditions (ICD-10), require the presence of both inattentive and hyperactive-impulsive symptoms ^8, 9^.

In 1994, the introduction of the DSM Fourth Edition (IV) ^10^ marked a diversion from this route by allowing for a diagnosis of ADHD when either hyperactive-impulsive *or* inattentive behaviors were present, and thereby defined three subtypes of ADHD: a) a predominantly inattentive type, b) a predominantly hyperactive-impulsive type, and c) a combined type ^11^. The following years saw a significant amount of research in which the importance of these subtypes in a clinical and epidemiological context was debated. For example, the clinical response to pharmacologic treatment by subtype or symptom clusters was investigated ^12– 14^, as were subtype differences in psychosocial functioning ^15, 16^, and the rate of comorbidity ^17^ in different age groups.

In both pediatric and adult populations, ADHD is significantly comorbid with a wide range of other DSM-IV disorders, irrespective of subtype. The most prevalent of these are mood, anxiety, impulse control, and substance use disorders (SUD) ^18– 20^. Data that stem primarily from clinical and population-based studies suggest that up to 89% of all adults with ADHD suffer from a psychiatric comorbidity during their lifetime ^16^, and that the comorbidity of SUD in adolescents and adults with ADHD might range from 16%–79% ^16, 21– 23^. The heterogeneity of these data is also reflected in research focused on the association between specific ADHD subtypes and SUDs. While some authors find no evidence of such an association, others have concluded that the hyperactive-impulsive subgroup is more likely to suffer from a comorbid SUD than is the inattentive subgroup ^15, 22, 24^.

To our knowledge, few studies have reported on the relationship between ADHD subtypes and SUD in adult samples. Furthermore, the limited data available stems primarily from America, while the few European studies focused on several comorbid factors, not solely on SUD ^16, 25^. The purpose of this study was therefore to characterize a clinical sample of adults with ADHD and to identify possible associations between ADHD subtypes, lifetime substance use, and preferences for specific substances.

## Methods

### Sample

Out of all consecutive referrals to the ADHD consultation service of the Zurich University Psychiatric Hospital ^26^ between 2002 and 2011, we included adults with a confirmed diagnosis of ADHD and with available information on substance use (N=413). There were no other inclusion or exclusion criteria.

### Assessment of ADHD symptomatology

The diagnosis of ADHD was based on the Utah criteria for diagnostic assessment with the Wender Reimherr Interview (WRI) ^27^, and translated into German and validated for the German language by Rösler *et al.* and Retz-Junginger *et al.* ^28– 30^. The Wender-Reimherr Interview is the German version of the American Wender-Reimherr Adult Attention Deficits Disorders Scale (WRAADDS) for the assessment of adult ADHD. It allows a diagnosis of adult ADHD to be made. It contains seven scales for: attention difficulties, persistent motor hyperactivity, temper, affective lability, emotional overreactivity, disorganization, and impulsivity. Each scale is represented by 3–5 items. A sum score is formed per scale, and each scale has a diagnostic threshold. A diagnosis requires that sum scores for scales 1–2 must each exceed their threshold, and that for scales 3–7, 2 out of 5 sum scores must exceed their threshold. According to DSM-IV Text Revision (TR) ^31^ specifications, three ADHD subtypes were identified: a predominantly inattentive subtype, a predominantly hyperactive-impulsive subtype, and a combined subtype. Subtypes were derived from the Attention Deficit-/Hyperactivity Self-Report Scale (ADHS-SB) questionnaire (see Supplementary material). The ADHS-SB is a self-rating instrument for the assessment of adult ADHD in German. It consists of 18 symptoms of ADHD derived from the DSM-IV and ICD-10 criteria for ADHD. The degree of endorsement is rated on four levels: 0 = not at all, 1 = slightly, 2 = moderately, and 3 = severely. The total score is obtained by summing up the 18 individual item scores. Subtype scores were obtained by first summing the respective items (items 1–9 for “inattentive”, items 10–18 for “hyperactive-impulsive”). Then, a cut-off value of 6 had to be exceeded in order for the respective subtype to be assigned. Subjects exceeding the threshold for both the inattentive and hyperactive-impulsive type were assigned to the combined subtype. Note that not all subjects fulfilled subtype criteria. The total number of subjects with a subtype assignment was 327. As reported elsewhere ^32^, patients also received a number of questionnaires, including German versions of the Symptom Check List 90-Revised (SCL-90-R) ^33^, the Wender Utah Rating Scale (WURS-k) ^28^, and the ADHS-SB ^34^. If patients did not answer all questions on the questionnaire items, they were approached again and asked to supply the missing information. When patients had difficulty answering a question, their therapist helped to clarify it and enable them to provide an answer. In addition, third-party information was sought from family members, spouses, school reports, and childhood medical reports to support the diagnostic procedure.

### Assessment of substance use and comorbidity

Assessment of substance use was based on ICD-10 criteria (F10-F19) ^9^. Subjects reported on the lifetime use of alcohol, opioids, cannabinoids, sedatives, cocaine, (non-cocaine) stimulants, hallucinogens, and tobacco. ICD-10 criteria were applied by a highly experienced clinician (DE) in a semi-structured interview. No official instrument was used. Substance use was differentiated into abuse/dependence and sub-threshold, i.e. non-dependent and non-abusive, but more-than-singular, use. Comorbid disorders were diagnosed according to ICD-10 by DE in a semi-structured interview, again without an official instrument.

### Statistical analysis

Fisher’s exact tests were used to compare frequency of substance abuse/dependence and comorbidity rates between ADHD subtypes, since small cell sizes were frequent. Kruskal-Wallis tests were used to compare questionnaire scores. Bonferroni correction was applied to all substance-related significance tests. A total of 26 tests were conducted, resulting in a Bonferroni-corrected significance threshold of p ≤.002. P-values surviving this threshold are printed in boldface in the results section. The study has low power: assuming a power of 80%, the minimal detectable difference in substance use frequency among subtypes is between 25–36%, while the power to detect a difference of 10% ranges from 28–48%. Analyses were carried out in Stata 11.2 and Stata 13.1 ^35^.

### Ethical framework

Authorization by the local ethics committee (Cantonal Ethics Committee Zurich; Kantonale Ethik Kommission Zürich (KEK)) was obtained before the study was conducted (04/2005). All participants received a written description of the study procedure and signed a consent form.

## Results

A total of 64 subjects had no questionnaire data whatsoever and were dropped from further analysis. These “drop-outs” were compared with the remaining 349 subjects and found not to differ in age and gender distribution. Drop-outs more often had affective disorders (24.9% vs. 12.7%, p=.05). They tended to have less overall substance abuse or dependence (14.1% vs. 27.8%, p=.02). Total substance use excluding abuse and dependence was clearly lower in drop-outs (23.4% vs. 63.6%, p=.000).

The average age of the included sample was 38.7 years (SD = 11.28), with a gender distribution that was 56% male and 44% female. Other than substance use, the most common comorbidities included affective disorders (25%); neurotic, stress-related and somatoform disorders (15%); and personality disorders (6%).

In the sample with questionnaire data (N=332–345, depending on questionnaire participants reached average test scores of 35.4 (SD=14.51) on WURS-k, 28.5 (SD=9.77) on ADHS-SB and 17.6 (SD=7.87) on the newly developed SCL-ADHD scale ^18^. A total of 233 subjects were identified as belonging to the combined subtype of ADHD (test scores: ADHS-SB 32.9 [SD=7.69], WURS-k 37.5 [SD=13.91], SCL-ADHD 19.4 [SD=7.62]), 70 belonged to the predominantly inattentive type (test scores: ADHS-SB 20.7 [SD=5.57], WURS-k 30.1 [SD=13.59], SCL-ADHD 14.2 [SD=6.52]), and 24 belonged to the predominantly hyperactive-impulsive type (test scores: ADHS-SB 23.9 [SD=6.68], WURS-k 40.8 [SD=16.16], SCL-ADHD 16.4 [SD=7.28]). WURS-k (p<.04) and ADHS-SB (p<.0001) scores were different between inattentive and hyperactive-impulsive subtypes, while all scores were different at p<.004 for the comparison of inattentive vs. combined subtype.

According to ICD-10 F1x, 26% of all participants at the time of the study, regardless of subtype, fulfilled the criteria for abuse of or dependence on psychotropic substances other than nicotine. The most frequently misused substances consisted of alcohol (8.9%), opioids (6.0%), cannabinoids (8.3%), and cocaine (8.0%). Nicotine abuse/dependence was found in 20.3% of participants.

Subtype-specific analyses revealed that 36.9% of the combined subgroup, 44.3% of the predominantly inattentive subgroup, and 41.7% of the hyperactive-impulsive subgroup currently suffered from a comorbid psychiatric disorder. Additionally, 31.3% of the combined-type individuals, 15.7% of the predominantly inattentive subjects and 41.7% of hyperactive-impulsive patients were diagnosed with abuse or dependence on a psychotropic substance other than nicotine. Table 1 summarizes the results.

**Table 1.** Lifetime substance use by ADHD subtype (p-values surviving Bonferroni threshold [p ≤ .002] in boldface).

	Inattentive type	Hyperactive- impulsive type ^a^	Combined type	p ^b^ inatt-hyp	p ^b^ inatt- combined
					
N	70	24	233		
					
	%	%	%		
Nicotine abuse/dependence	12.9	12.5	24.9	1.0	.03
Opiates abuse/dependence	1.4	12.5	7.3	.05	.08
Stimulants abuse/dependence	7.1	4.2	9.4	1.0	.64
Alcohol abuse/dependence	1.4	8.3	11.2	.16	.01
Cannabis abuse/dependence	5.7	16.7	8.6	.20	.61
Cocaine abuse/dependence	0	12.5	10.3	.02	**.002**
Substances total (w/o tobacco) abuse/dependence	15.7	41.7	31.3	.02	.01
Substances total (w/o tobacco) use	57.1	62.5	66.5	.81	.16

^b^Fischer's exact test

inatt-hyp = inattentive vs. hyperactive-impulsive subtype, inatt-combined = inattentive vs combined subtype

## Discussion

The present study investigated associations between the combined and predominantly inattentive subtypes of adults with ADHD and lifetime substance use, within a clinical sample. The most clinically significant result is the finding that the inattentive subtype showed a statistically significantly smaller rate of cocaine abuse/dependence compared to the combined subtype.

These results are in line with earlier work by Sobanski *et al.*, who had characterized a sample of 118 adults with ADHD and found that the combined type suffered significantly more from lifetime SUDs (48.4%) than did patients with a predominantly inattentive type (23.3%) ^16^. On the other hand, our findings contrast with results published by Clure *et al.*, who reported on 43 patients with adult ADHD but found no differences in ADHD subtypes when divided by substance of choice (cocaine, alcohol, and multiple substances) ^36^.

The most frequently consumed substance among all study participants was nicotine. This finding is in accord with results from prior studies ^37– 39^. With regard to subtype-specific differences, some authors have reported that, at least in young adolescents, the inattentive subtype of ADHD is more likely to correlate with higher levels of nicotine use than does the combined subtype ^40^. It was suggested that nicotine might primarily improve attention but have less influence on hyperactive-impulsive behavior, which might explain this finding ^41, 42^. Other researchers, however, suggest that hyperactive-impulsive symptoms present a greater risk for frequent nicotine use than do inattentive symptoms at a later age, and argue that the relationship between ADHD symptoms and nicotine use might change between adolescence and adulthood ^43^.

Our hypothesis that findings would show continuing preferences for the use of specific substances in adulthood according to subtype (beyond cocaine), remains open due to lack of statistical significance. Like earlier reports of (non-cocaine) stimulants being used as self-medication by patients with ADHD, we had also expected to find a higher rate of non-prescribed lifetime stimulant abuse/dependence in the hyperactive-impulsive type, but not in the inattentive one ^44, 45^. In this sample, however, we found no evidence for this assumption, but lack of statistical power precludes interpreting this as evidence of no difference. We suspect that adults with both hyperactive-impulsive and inattentive symptoms might initially prefer cocaine to stimulants for self-medication, but there is no direct evidence for this assessment ^46– 48^.

The possibility of using cocaine as an attempt to self-medicate for ADHD symptoms was originally proposed in the early ’90s ^49, 50^. More recently, Saules *et al.* compared the symptom profile among adult ADHD smokers with and without cocaine dependence, and found that when they corrected for the use of nicotine, adults who used cocaine exhibited a more severe adult ADHD symptom profile, as accounted for by the presence of elevated hyperactive-impulsive but not inattentive symptoms. He therefore suggested that cocaine use in smokers with ADHD might be driven by excesses in hyperactivity ^50^. Despite differences in sampling, our results are in accord with this finding.

On a different note van Wingen et al investigated structural brain abnormalities in this population and reported of significantly smaller grey matter volumes in the occipital cortex as well as smaller volumes in the putamen in ADHD patients with comorbid cocaine dependence when compared to those without this lifetime diagnosis. The authors of aforementioned study suggested that the differences in putamen volumes may reflect alterations in the availability of striatal dopamine transporters that are available for interaction with methylphenidate, thus giving some explanation for the finding that methylphenidate is less effective in patients with ADHD and a comorbid cocaine dependence ^51^.

The main limitation of this study is low power. This means, in particular, that non significant findings cannot be interpreted as evidence of no difference. A further limitation is that our sample was recruited entirely within a university setting, which might contribute to a selection bias. As a result, this clinical sample might have different characteristics than patients would exhibit who are in treatment with a physician in private practice. Nevertheless, the ADHD consultation service of the Psychiatric University Hospital Zurich is the largest institution of its kind in Switzerland and attracts patients from diverse psychosocial backgrounds. Furthermore comorbidities, particularly personality disorders might have confounded the results. For instance, Borderline personality disorder, which often co-occurs with ADHD and is difficult to differentiate, is also known to be associated with SUD ^52^. However in this sample that relied for diagnosis of comorbidity on a semi-structured clinical interview, but not on additional instruments, we found only 6% of patients suffering from a comorbid personality disorder. This is low in comparison to some studies reporting prevalence rates between 25 – 78% ^53– 57^.

In conclusion, our findings underscore the high rate of comorbidity between substance use and ADHD in adults. The more frequent abuse/dependence of cocaine by adult patients with hyperactive-impulsive symptoms should be kept in mind when treating this patient group. Although a limited number of evidence-based treatment strategies currently exist for the concurrent treatment of ADHD and SUD, some studies suggest that stimulant medication remains an efficacious pharmacological treatment option that improves symptoms of ADHD without increasing the likelihood of relapse into SUD ^22, 58^. Furthermore a study among patients with ADHD and a comorbid cocaine dependence receiving methylphenidate, demonstrated an advantage over placebo with regard to reduction in cocaine use in individuals who responded to ADHD treatment ^59, 60^.

## Data availability

The data referenced by this article are under copyright with the following copyright statement: Copyright: © 2016 Liebrenz M et al.

ZENODO: Dataset 1. Contains all the variables necessary to reproduce the results of Adult attention-deficit/hyperactivity disorder: Associations between subtype and lifetime substance use – a clinical study, Liebrenz *et al.,* doi: 10.5281/zenodo.19623 ^61^

ZENODO: Stata source code to reproduce analysis, doi: 10.5281/zenodo.19622 ^62^

## Consent

Written informed consent was obtained from patients.

## Supplementary material

**The ADHS-SB questionnaire (German).**
