# Supplementary material for: Adult attention-deficit/hyperactivity disorder: Associations between subtype and lifetime substance use – a clinical study
Source: F1000Res. 2016 Oct 19;4:407. Originally published 2015 Jul 28. [Version 2] doi: 10.12688/f1000research.6780.2 (PMC5089146; doi:10.12688/f1000research.6780.2)
Supplement: Supplementary file 1 [file f1000research-4-10511-s0000.tgz › b22ad5bb-1f38-461a-bc07-d683e224dbd2.pdf]

# A6 ADHS-Selbstbeurteilungsskala (ADHS-SB) (Rösler et al. 2004)

**ADHS-SB**

Code ........................................

Geboren am: ................................

Nachfolgend finden Sie einige Fragen über Konzentrationsvermögen, Bewegungsbedürfnis und Nervosität. Gemeint ist damit Ihre Situation, wie sie sich gewöhnlich darstellt.

Wenn die Formulierungen auf Sie nicht zutreffen, kreuzen Sie bitte »nicht zutreffend« an. Wenn Sie der Meinung sind, dass die Aussagen richtig sind, geben Sie bitte an, welche Ausprägung – leicht/mittel/schwer – Ihre Situation am besten beschreibt.

| | |
|---|---|
| 0 | trifft nicht zu |
| 1 | leicht ausgeprägt (kommt gelegentlich vor) |
| 2 | mittel ausgeprägt (kommt oft vor) |
| 3 | schwer ausgeprägt (kommt nahezu immer vor) |

Bitte kreuzen Sie die entsprechende Antwortalternative an. Lassen Sie bitte keinen Punkt aus.

Beispiel:

Ich bin unaufmerksam gegenüber Details oder mache Sorgfaltsfehler bei der Arbeit. ☐ 0 ☐ 1 ☐ 2 ☒ 3

In diesem Fall ist die 3 (»schwer ausgeprägt«) angekreuzt: Das würde bedeuten, dass Sie stark ausgeprägt und nahezu immer Aufmerksamkeitsprobleme haben.

| | | | | |
|---|---|---|---|---|
| 1. Ich bin unaufmerksam gegenüber Details oder mache Sorgfaltsfehler bei der Arbeit. | ☐ 0 | ☐ 1 | ☐ 2 | ☐ 3 |
| 2. Bei der Arbeit oder sonstigen Aktivitäten (z. B. Lesen, Fernsehen, Spiel) fällt es mir schwer, konzentriert durchzuhalten. | ☐ 0 | ☐ 1 | ☐ 2 | ☐ 3 |
| 3. Ich höre nicht richtig zu, wenn jemand etwas zu mir sagt. | ☐ 0 | ☐ 1 | ☐ 2 | ☐ 3 |
| 4. Es fällt mir schwer, Aufgaben am Arbeitsplatz, wie sie mir erklärt wurden, zu erfüllen. | ☐ 0 | ☐ 1 | ☐ 2 | ☐ 3 |
| 5. Es fällt mir schwer, Projekte, Vorhaben oder Aktivitäten zu organisieren. | ☐ 0 | ☐ 1 | ☐ 2 | ☐ 3 |

| | | | | |
|---|---|---|---|---|
| 6. Ich gehe Aufgaben, die geistige Anstrengung erforderlich machen, am liebsten aus dem Weg. Ich mag solche Arbeiten nicht oder sträube mich innerlich dagegen. | ☐ 0 | ☐ 1 | ☐ 2 | ☐ 3 |
| 7. Ich verlege wichtige Gegenstände (z. B. Schlüssel, Portemonnaie, Werkzeuge). | ☐ 0 | ☐ 1 | ☐ 2 | ☐ 3 |
| 8. Ich lasse mich bei Tätigkeiten leicht ablenken. | ☐ 0 | ☐ 1 | ☐ 2 | ☐ 3 |
| 9. Ich vergesse Verabredungen, Termine oder telefonische Rückrufe. | ☐ 0 | ☐ 1 | ☐ 2 | ☐ 3 |
| 10. Ich bin zappelig. | ☐ 0 | ☐ 1 | ☐ 2 | ☐ 3 |
| 11. Es fällt mir schwer, längere Zeit sitzen zu bleiben (z. B. im Kino, Theater). | ☐ 0 | ☐ 1 | ☐ 2 | ☐ 3 |
| 12. Ich fühle mich unruhig. | ☐ 0 | ☐ 1 | ☐ 2 | ☐ 3 |
| 13. Ich kann mich schlecht leise beschäftigen. Wenn ich etwas mache, geht es laut zu. | ☐ 0 | ☐ 1 | ☐ 2 | ☐ 3 |
| 14. Ich bin ständig auf Achse und fühle mich wie von einem Motor angetrieben. | ☐ 0 | ☐ 1 | ☐ 2 | ☐ 3 |
| 15. Mir fällt es schwer abzuwarten, bis andere ausgesprochen haben. Ich falle anderen ins Wort. | ☐ 0 | ☐ 1 | ☐ 2 | ☐ 3 |
| 16. Ich bin ungeduldig und kann nicht warten, bis ich an der Reihe bin (z. B. beim Einkaufen). | ☐ 0 | ☐ 1 | ☐ 2 | ☐ 3 |
| 17. Ich unterbreche und störe andere, wenn sie etwas tun. | ☐ 0 | ☐ 1 | ☐ 2 | ☐ 3 |
| 18. Ich rede viel, auch wenn mir keiner zuhören will. | ☐ 0 | ☐ 1 | ☐ 2 | ☐ 3 |
| 19. Diese Schwierigkeiten hatte ich schon im Schulalter. | ☐ 0 | ☐ 1 | ☐ 2 | ☐ 3 |
| 20. Diese Schwierigkeiten habe ich immer wieder, nicht nur bei der Arbeit, sondern auch in anderen Lebenssituationen, z. B. Familie, Freunde und Freizeit. | ☐ 0 | ☐ 1 | ☐ 2 | ☐ 3 |
| 21. Ich leide unter diesen Schwierigkeiten. | ☐ 0 | ☐ 1 | ☐ 2 | ☐ 3 |
| 22. Ich habe wegen dieser Schwierigkeiten schon Probleme im Beruf und auch im Kontakt mit anderen Menschen gehabt. | ☐ 0 | ☐ 1 | ☐ 2 | ☐ 3 |

Bitte prüfen Sie, ob Sie alle Fragen beantwortet haben.

---

$\sum$ Item 1–9: inattentive

$\sum$ Item 10–14: hyperactive

$\sum$ Item 15–18: impulsive

$\sum$ Item 1–18: ……………………

Ergebnis der Auswertung

DIAGNOSE: ☐ 314.00 (DSM-IV)

☐ 314.01 (DSM-IV)

☐ F90.0 (ICD-10)

---

(nur vom Untersucher auszufüllen)

### *ADHS-SB, Auswertung*

*Zur Diagnose einer ADHS werden gemäß der ICD-10-Forschungskriterien 6 positive Merkmale aus dem Bereich 1–9 benötigt sowie 3 der Merkmale 10–14 und ein Merkmal der Items 15–18. Nach DSM-IV müssen von den Items 1–9 sechs positiv sein (Score >0) sowie von den Merkmalen 10–18 weitere 6 Items.*

*Als positiv wird ein Merkmal dann gewertet, wenn seine Ausprägung >0 beträgt. Da weder DSM noch ICD eine Quantifizierung der Merkmale enthalten, genügt für die Einschätzung eines Merkmals als vorhanden ein Wert größer 0. (Manchmal kann es im wissenschaftlichen Bereich sinnvoll sein, die Schwelle für die Bejahung eines Merkmals zu erhöhen und als Mindestausprägung z. B. mittel (2) zu verlangen. Für den Klinik- und Praxisalltag genügt aber die Regel von ICD-10 und DSM-IV, dass ein Merkmal vorhanden ist, selbst wenn die Ausprägung des Merkmals nur leicht ist.)*
