# Supplementary material for: Adult attention-deficit/hyperactivity disorder: Associations between subtype and lifetime substance use – a clinical study
Source: F1000Res. 2016 Oct 19;4:407. Originally published 2015 Jul 28. [Version 2] doi: 10.12688/f1000research.6780.2 (PMC5089146; doi:10.12688/f1000research.6780.2)
Supplement: Peer review history

# Peer review: Referee response for version 1

**DOI:** 10.5256/f1000research.7284.r17021

**Published:** 2016-10-17

**Authors:** Joseph O'Neill

**Affiliations:** 1 Division of Child and Adolescent Psychiatry, UCLA Semel Institute for Neuroscience and Human Behavior, Los Angeles, CA, USA

The investigators determined the effect of ADHD subtype on the prevalence of substance use disorders (SUDs) in a large (413 patients) sample of (medicated?) adult ADHD. In line with expectations, the overall sample evinced high rates of lifetime substance abuse or dependence (26%) and occasional use (57%). The inattentive subtype was significantly less likely to abuse or be dependent on cocaine than the combined subtype. As indicated by the Bordelais reviewers, while not a major advance, this study is a perfect example of normal science in the sense of Kuhn and represents a worthwhile gain in our knowledge of ADHD.

1. The authors list multiple clinical scales used to assess ADHD and state that SUD was assessed based on ICD-10 criteria. Like the Freiburger reviewers, the first question that occurred to me was whether any standardized instrument was administered to assess SUDs?
2. Again, as asked by other reviewers, were the patients in the sample undergoing current pharmacological treatment? Presumably, many were on methylphenidate or other agents.
3. It would be a good idea to cite some statistics for prevalence substance abuse and dependence in the general population around Zürich. Then the reader would have an idea how much higher prevalence is in the adult ADHD clinical sample.
4. The investigators were commendably conservative in claiming significant results, restricting themselves to *p*-values ≤ 0.002, based on Bonferroni correction for multiple comparisons. It might be informative to discuss some of the other results that would have been significant under a less stringent cut-off. For example, nicotine abuse/dependence is twice as frequent and alcohol abuse/dependence is nearly ten times as frequent in the Combined as in the Inattentive subgroup. These may be worth commenting on. But I wouldn’t discuss any comparisons involving the Hyperactive-Impulsive subgroup, since they are too few (only 24 subjects).
5. In the Introduction, the authors retell the diagnosis and subtyping of ADHD in DSM-III-R, ICD-10, and DSM-IV. They should add a line or two about DSM5, just to round out their brief historical review.

I have read this submission. I believe that I have an appropriate level of expertise to confirm that it is of an acceptable scientific standard.

# Peer review: Referee response for version 1

**DOI:** 10.5256/f1000research.7284.r16482

**Published:** 2016-09-21

**Authors:** Swantje Matthies, Eliza Hoxhaj

**Affiliations:** 1 Department of Psychiatry and Psychotherapy, University Medical Center Freiburg, Freiburg, Germany

The paper "Adult attention-deficit/hyperactivity disorders: Associations between subtype and lifetime substance use – a clinical study” covers, besides the well-known comorbidity of ADHD with substance use disorders (SUDs), a very interesting field of research concerning the associations between ADHD subtypes and substance use disorders. It adds to the knowledge and differentiates knowledge in focusing on ADHD subtypes. Particularly we noted the large sample size, as it allows more reliable results, the valid assessment of ADHD by using standardized instruments and third-party information and the well detailed statistical analysis. The applied methods appear overall reasonable and valid to us.

 We have some remarks that should be addressed:

1.  The authors mentioned that assessment of substance use was based on ICD-10 criteria. However they didn’t exactly explain their method to gather this data. We noted here the lack of standardized instruments. Furthermore they pooled substance abuse and dependence in one category. The rational for using this procedure should be given in more detail.
2. We missed information about psychopharmacological treatment. This information might provide potential correlations of treatment/no treatment with substance use.
3. Another question regards the comorbid disorders: It should be discussed that comorbidities, particularly personality disorders might have confounded the results. For instance, Borderline personality disorder, which often co-occurs with ADHD ^1^ is also known to co-occur with SUD. Further, we wonder that only 6% of the patients had a comorbid personality disorder, which is low in contrast to some studies reporting prevalence rates of personality disorders between 25% and 78% among individuals with ADHD ^2^^-^^5^.  It is also important to make clear, how the authors assessed comorbid disorders. Did they use standardized instruments?
4. The sample size of 327 represented on Table 1, page 4, does not correspond to the described sample size in the paragraph above “Results” (“…were compared with the remaining 349 subjects…” or “N=332-345, depending on questionnaire participants…”).
5.  In the discussion it might be interesting to explain the use and effects of cocaine among individuals with ADHD, especially with regard to neurobiology. It would also be interesting to know about possible hypotheses and assumptions among the authors concerning the subtype difference in cocaine abuse/dependence. Are there neuropsychological or clinical differences between the subtypes which protect the inattentive subtype from cocaine dependence?
6. The discussion should also comment on the relevance of the presented findings for therapeutic approaches.

We have read this submission. We believe that we have an appropriate level of expertise to confirm that it is of an acceptable scientific standard, however we have significant reservations, as outlined above.

## References

[1] PhilipsenALimbergerMFLiebKFeigeBKleindienstNEbner-PriemerUBarthJSchmahlCBohusM: Attention-deficit hyperactivity disorder as a potentially aggravating factor in borderline personality disorder. *Br J Psychiatry*.2008;192(2) : 10.1192/bjp.bp.107.035782 118-23 10.1192/bjp.bp.107.035782 18245028

[2] JacobCPRomanosJDempfleAHeineMWindemuth-KieselbachCKruseAReifAWalitzaSRomanosMStrobelABrockeBSchäferHSchmidtkeABöningJLeschKP: Co-morbidity of adult attention-deficit/hyperactivity disorder with focus on personality traits and related disorders in a tertiary referral center. *Eur Arch Psychiatry Clin Neurosci*.2007;257(6) : 10.1007/s00406-007-0722-6 309-17 10.1007/s00406-007-0722-6 17401730

[3] CumynLFrenchLHechtmanL: Comorbidity in adults with attention-deficit hyperactivity disorder. *Can J Psychiatry*.2009;54(10) :673-83 1983567410.1177/070674370905401004

[4] MatthiesSvan ElstLTFeigeBFischerDScheelCKrogmannEPerlovEEbertDPhilipsenA: Severity of childhood attention-deficit hyperactivity disorder--a risk factor for personality disorders in adult life?. *J Pers Disord*.2011;25(1) : 10.1521/pedi.2011.25.1.101 101-14 10.1521/pedi.2011.25.1.101 21309626

[5] WilliamsEDReimherrFWMarchantBKStrongREHallsCSoniPGalePDRobisonRJ: Personality disorder in ADHD Part 1: Assessment of personality disorder in adult ADHD using data from a clinical trial of OROS methylphenidate. *Ann Clin Psychiatry*.2010;22(2) :84-93 20445835

# Response

**Published:** 2016-10-04

**Authors:** Michael Liebrenz

**Affiliations:** University of Bern, Institute of Legal Medicine, Department of Forensic Psychiatry, Switzerland

We appreciate the dedicated and useful comments of Dr. Swantje and Dr. Hoxhaj and the editor

 and the chance they gave us to improve the manuscript.

  

1. The reviewer asks us to elaborate on how ICD-10 criteria for substance use were assessed and why abuse and dependence were pooled.
  1. ICD-10 criteria were applied by a highly experienced clinician (DE) in a semi-structured interview. No official instrument was used.
  2. Estimates of percentages would have been small  and noisy if we had separated dependence and abuse. Combining them gives a more robust estimate and seems justified to us also because both describe a serious level of substance use that requires treatment.
2. The reviewer asks for information on psychopharmacological treatment. Unfortunately, such information is not available.
3. The reviewer asks us to discuss and to clarify assessment of comorbidities.
  We have addressed these questions in the Methods and in the Discussion (Limitations) Section. We now state: *“Comorbid disorders were diagnosed according to ICD-10 by DE in a semi-structured interview, again without an official instrument.” and “Furthermore comorbidities, particularly personality disorders might have confounded the results. For instance, Borderline personality disorder, which often co-occurs with ADHD and is difficult to differentiate, is also known to be associated with SUD. However in this sample that relied for diagnosis of comorbidity on a semi-structured clinical interview, but not on additional instruments, we found only 6% of patients suffering from a comorbid personality disorder. This is low in comparison to some studies reporting prevalence rates between 25 – 78%.”*
4. The  N=327 reflects the fact that not all patients diagnosed with ADHD fulfilled subtype criteria. Remember that in this study, ADHD was diagnosed according to Utah (Wender-Rheimherr), not DSM, criteria, and subtypes were constructed post-hoc using scores on the ADHS-SB questionnaire. This means that the diagnosis itself does not necessarily entail a subtype assignment, and vice versa. Thus,  in about 20 participants ADHS-SB scores were not high enough to yield a subtype classification. This is now explained in the methods section under the heading "Assessment of ADHD symptomatology".
5. The reviewer suggests to discuss the use and effects of cocaine among individuals with ADHD, especially with regard to neurobiology. We have addressed these aspects in an expanded discussion section. We now state: “ *On a different note van Wingen et al investigated structural brain abnormalities in this population and reported of significantly smaller grey matter volumes in the occipital cortex as well as smaller volumes in the putamen in ADHD patients with comorbid cocaine dependence when compared to those without this lifetime diagnosis. The authors of aforementioned study suggested that the differences in putamen volumes may reflect alterations in the availability of striatal dopamine transporters that are available for interaction with methylphenidate, thus giving some explanation for the finding that methylphenidate is less effective in patients with ADHD and a comorbid cocaine dependence.”*
6. The reviewer asks us to comment on the relevance of the presented findings for therapeutic approaches. We now point towards research by Levin et al and have expanded the conclusion section: “ *Furthermore a study among patients with ADHD and a comorbid cocaine dependence receiving methylphenidate, demonstrated an advantage over placebo with regard to reduction in cocaine use in individuals who responded to ADHD treatment.”*

# Peer review: Referee response for version 1

**DOI:** 10.5256/f1000research.7284.r11890

**Published:** 2016-02-17

**Authors:** Marc Auriacombe, Jean-Marc Alexandre, Melina Fatseas

**Affiliations:** 1 Addiction Psychiatry Team, Sanpsy CNRS USR 3413, University of Bordeaux, Bordeaux, France; 2 Pôle Addictologie, CH Ch. Perrens / CHU Bordeaux, Bordeaux, France

In this clinical study, Liebrenz *et al*. addressed the association between Adult attention-deficit/hyperactivity disorder (ADHD) subtypes and lifetime substance-use disorders (SUD). This topic is of particular interest (Fatseas *et al* 2012), as there is a strong literature supporting a relationship between ADHD and SUD (Wilens 2004). An emerging issue is the relationship between SUD and ADHD subtypes (Inattentive, Hyperactive-Impulsive, and Combined). For instance, Tamm *et al*. found more severe SUDs for the Combined subtype (Tamm *et al*. 2012). Our team suggested that Combined and Inattentive subtypes might be associated with more severe addictive disorders (Hurmic *et al*., CPDD annual meeting 2015). As stated in the manuscript, other authors reported the Hyperactive-impulsive subtype more likely to be associated to a comorbid SUD, or no difference. Data remains limited and this topic is still in debate.

In this perspective, Liebrenz *et al*. conducted a cross sectional study with validated questionnaires assessing ADHD symptomatology and substance use, among 413 adults diagnosed with ADHD. They hypothesized that results would show statistical differences of lifetime SUD prevalence for specific substances, according to ADHD subtype. The main finding of this study was that the Inattentive ADHD subtype was significantly less associated than the Combined subtype to lifetime cocaine abuse/dependence (there was no difference for other substances). However, Hyperactive-impulsive and Inattentive subtypes were not statistically different for their association to any substance disorder.

Liebrenz *et al*.’s work is a significant contribution – but not a breakthrough – to research on the relationships of ADHD subtypes and addiction. As reviewers, we noted the clarity of their manuscript, the justification of the study and the definition of their purpose in the introduction section. The method seemed valid to us and missing data (64 subjects) were correctly managed. However, we think that the strategy of analysis should have been more detailed in a specific paragraph. For instance, the usefulness of the WURS-K questionnaire is not explained. The Wender Reimherr Interview (WRI) and the Attention Deficit-/Hyperactivity Self-Report Scale (ADHS-SB) would also have deserved a short presentation and explanation of their use in this study. Another point to argue is the choice of the WRI to diagnose ADHD, as Rosler *et al*. (2006) stated that “on the basis of the WRI, DSM-IV diagnoses cannot be made”, even if “considerable amount of overlap exists” (Rosler *et al*. 2006).

Results are presented and discussed appropriately. The authors mentioned that assessment of substance use was based on ICD-10 criteria. However, for their analysis they pooled substance abuse and dependence in one category without giving the rationale for that choice. In ICD-10 and DSM-IV these are distinct entities. However, in DSM 5 these distinct categories have been combined with other adjustments into one unique category (Hasin *et al* 2013). The authors might clarify this use of ICD-10 in a DSM 5 perspective. The ICD-10 substance use categories might have allowed a more specific study of the association of addiction and ADHD-subtypes?

The absence of statistical difference between Hyperactive-impulsive and Inattentive subtypes for association with any substance disorder was unexpected, especially for stimulants. Liebrenz *et al*. discussed the lack of statistical power of their study, which could explain the lack of significant differences. Thus, the hypothesis of a link between specific SUDs and specific ADHD subtypes remains open.

In conclusion, the study of Liebrenz *et al*. illustrates the high rate of comorbidity between substance use disorders and ADHD in adults, and reports a specific relationship between cocaine disorder and ADHD Combined subtype. Further research with more statistical power may highlight other specificities of ADHD subtypes in the field of addiction.

We have read this submission. We believe that we have an appropriate level of expertise to confirm that it is of an acceptable scientific standard.

## References

[1] FatseasMDebrabantRAuriacombeM: The diagnostic accuracy of attention-deficit/hyperactivity disorder in adults with substance use disorders. *Curr Opin Psychiatry*.2012;25(3) : 10.1097/YCO.0b013e3283523d7c 219-25 10.1097/YCO.0b013e3283523d7c 22449768

[2] HurmicHDebrabantRKervranCSerreFAuriacombeMFatseasM: ADHD and substance use disorders: Subtype and gender differences. *Drug and Alcohol Dependence*.2015;156: 10.1016/j.drugalcdep.2015.07.1190 e100-e101 10.1016/j.drugalcdep.2015.07.1190

[3] TammLAdinoffBNakoneznyPAWinhusenTRiggsP: Attention-deficit/hyperactivity disorder subtypes in adolescents with comorbid substance-use disorder. *Am J Drug Alcohol Abuse*.2012;38(1) : 10.3109/00952990.2011.600395 93-100 10.3109/00952990.2011.600395 21834613PMC3441182

[4] RöslerMRetzWThomeJSchneiderMStieglitzRDFalkaiP: Psychopathological rating scales for diagnostic use in adults with attention-deficit/hyperactivity disorder (ADHD). *Eur Arch Psychiatry Clin Neurosci*.2006;256 Suppl 1: 10.1007/s00406-006-1001-7 i3-11 10.1007/s00406-006-1001-7 16977549

[5] HasinDSO'BrienCPAuriacombeMBorgesGBucholzKBudneyAComptonWMCrowleyTLingWPetryNMSchuckitMGrantBF: DSM-5 criteria for substance use disorders: recommendations and rationale. *Am J Psychiatry*.2013;170(8) : 10.1176/appi.ajp.2013.12060782 834-51 10.1176/appi.ajp.2013.12060782 23903334PMC3767415

[6] WilensTE: Attention-deficit/hyperactivity disorder and the substance use disorders: the nature of the relationship, subtypes at risk, and treatment issues. *Psychiatr Clin North Am*.2004;27(2) : 10.1016/S0193-953X(03)00113-8 283-301 10.1016/S0193-953X(03)00113-8 15063998

# Response

**Published:** 2016-10-04

**Authors:** Michael Liebrenz

**Affiliations:** University of Bern, Institute of Legal Medicine, Department of Forensic Psychiatry, Switzerland

We appreciate the time and effort of Dr. Auriacombe, Dr. Alexandre and Dr. Fatseas and respond to their concerns below: 

 1.The reviewers ask us to explain the usefulness of the WURS-K questionnaire. The WURS-k retrospectively assesses symptoms of ADHD in childhood and as such is part of the diagnostic process for adult ADHD. It is mentioned here for completeness, not because it was a target of analysis. 

 2.The reviewers ask for more detailed presentations of the Wender Reimherr Interview (WRI) and the Attention Deficit-/Hyperactivity Self-Report Scale (ADHS-SB). We have now added the following two paragraphs on these tests:

 WRI: "The Wender-Reimherr Interview is the German version of the American Wender-Reimherr Adult Attention Deficits Disorders Scale (WRAADDS) for the assessment of adult ADHD. It allows a diagnosis of adult ADHD to be made. It contains seven scales for: attention difficulties, persistent motor hyperactivity, temper, affective lability, emotional overreactivity, disorganization, and impulsivity. Each scale is represented by 3–5 items. A sum score is formed per scale, and each scale has a diagnostic threshold. A diagnosis requires that sum scores for scales 1–2 must each exceed their threshold, and that for scales 3–7, 2 out of 5 sum scores must exceed their threshold."

 ADHS-SB: " The ADHS-SB is a self-rating instrument for the assessment of adult ADHD in German.  It consists of 18 symptoms of ADHD derived from the DSM-IV and ICD-10 criteria for ADHD. The degree of endorsement is rated on four levels: 0 = not at all, 1 = slightly, 2 = moderately, and 3 = severely. The total score is obtained by summing up the 18 individual item scores. Subsyndrome scores for ‘‘attention deficit’’, ‘‘hyperactivity’’, and ‘‘impulsivity’’ can also be obtained." 

 3. The reviewers question the choice of the WRI to diagnose ADHD, stating that according to Rosler et al. (2006), DSM-IV diagnoses cannot be made based on the WRI. This is true. However, the DSM-IV does not contain a specific diagnosis for *adult* ADHD, while the WRI is specifically targeted to adults. As Rosler also states "the question remains whether the DSM-IV criteria are adequate to characterize adult ADHD" (p. I/4) and "An advantage of the WRI could be the extended spectrum of adult psychopathology" (p. I/10). There is certainly room to argue for either diagnostic approach. Reassuringly, as the reviewers also note, the two approaches show considerable overlap. 

 4.The reviewers ask for the rationale for pooling substance abuse and dependence. The reason is primarily statistical: estimates of percentages would have been small and noisy if we had separated dependence and abuse. Combining them gives a more robust estimate. This also seems justified to us because both abuse and dependence describe a serious level of substance use that requires treatment.
